# Role of tube size and intranasal compression of the nasotracheal tube in respiratory pressure loss during nasotracheal intubation: a laboratory study

**DOI:** 10.1186/s12871-017-0432-1

**Published:** 2017-10-17

**Authors:** Koichi Futagawa, Yoshihiro Takasugi, Takeharu Kobayashi, Satoshi Morishita, Takahiko Okuda

**Affiliations:** 1 Department of Anesthesiology, Nara Hospital, Kindai University Faculty of Medicine, 1248-1 Otodacho, Ikoma, Nara, 630-0293 Japan; 2 Department of Anesthesiology, Kindai University Faculty of Medicine, 377-2 Ohno-higashi, Osaka-sayama, Osaka, 589-8511 Japan; 3 Product Development Research Center, Daiken Medical Co. Ltd., 2-6-2, Ayumino, Izumi-city, Osaka, 594-1157 Japan

**Keywords:** Endotracheal tube, Pressure loss, Slip joint, Turbulent flow, Nasotracheal intubation

## Abstract

**Background:**

Small nasotracheal tubes (NTTs) and intranasal compression of the NTT in the nasal cavity may contribute to increasing airway resistance. Since the effects of size, shape, and partial compression of the NTT on airway resistance have not been investigated, values of airway resistance with partial compression of preformed NTTs of various sizes were determined.

**Methods:**

To determine the factors affecting the respiratory pressure loss during the nasotracheal intubation, physical and fluid dynamics simulations were used. The internal minor axes of NTTs in the nasal cavity of intubated patients were measured using dial calipers. In physical and fluid dynamics simulations, pressure losses through the tubular parts, compressed parts, and slip joints of NTTs with internal diameters (IDs) of 6.0, 6.5, 7.0, 7.5, and 8.0 mm were estimated under partial compression.

**Results:**

The median internal minor axes of the 7.0- and 7.5-mm ID NTTs in the nasal cavity were 5.2 (4.3–5.6) mm and 6.0 (4.2–7.0) mm, respectively. With a volumetric air flow rate of 30 L/min, pressure losses through uncompressed NTTs with IDs of 6.0-, 6.5-, 7.0-, 7.5- and 8.0-mm were 651.6 ± 5.7 (6.64 ± 0.06), 453.4 ± 3.9 (4.62 ± 0.04), 336.5 ± 2.2 (3.43 ± 0.02), 225.2 ± 0.2 (2.30 ± 0.00), and 179.0 ± 1.1 Pa (1.82 ± 0.01 cmH_2_O), respectively; the pressure losses through the slip joints were 220.3 (2.25), 131.1 (1.33), 86.8 (0.88), 57.1 (0.58), and 36.1 Pa (0.37 cmH_2_O), respectively; and the pressure losses through the curvature of the NTT were 71.6 (0.73), 69.0 (0.70), 64.8 (0.66), 32.5 (0.33), and 41.6 Pa (0.42 cmH_2_O), respectively. A maximum compression force of 34.1 N increased the pressure losses by 82.0 (0.84), 38.0 (0.39), 23.5 (0.24), 16.6 (0.17), and 9.3 Pa (0.09 cmH_2_O), respectively.

**Conclusion:**

Pressure losses through NTTs are in inverse proportion to the tubes’ IDs; greater pressure losses due to slip joints, acute bending, and partial compression of the NTT were obvious in small NTTs. Pressure losses through NTTs, especially in small NTTs, could increase the work of breathing to a greater extent than that through standard tubes; intranasal compression further increases the pressure loss.

## Background

The size of the endotracheal tube is the major factor governing resistance in anesthesia breathing systems [1]. Clinically, the airway resistance through endotracheal tubes depends on the internal diameter and length of the tubes, and pressure loss through the slip joint accounts for a large percentage of the total pressure loss, especially through small endotracheal tubes: total pressure losses through standard endotracheal tubes of 6.0- and 7.0-mm internal diameter (ID) are greater by 280% and 80%, respectively, compared with an 8.0-mm ID tube [2].

Nasotracheal tubes (NTTs) tend to further increase pressure losses through the tubes as compared to orotracheal tubes, due to the longer than standard length of the tubes and creation of a sharp bend along the bridge of the nose when in use. Furthermore, during nasotracheal intubation, the NTT is compressed by the nasal septum and nasal concha in the tight nasal cavity. An internal minor axis (henceforth called minor axis) of the intranasal part of the NTT of less than 2.7–3.5 mm due to compression during nasotracheal intubation has been previously reported [3, 4]. Reduction in the cross-sectional area of a polyvinyl chloride (PVC) NTT in the nasal cavity may lead to further increase in airway resistance.

Endotracheal intubation increases the airway resistance of spontaneously breathing patients. The increased work of breathing secondary to the increase in airway resistance with endotracheal intubation is usually compensated during positive pressure mechanical ventilation. [5–7] With spontaneous ventilation at emergence from anesthesia or while weaning from mechanical ventilation in the intensive care unit, however, this increased work of breathing can have severe consequences, particularly in patients with compromised lung function [8].

For less traumatic passage of the tube, a NTT of a smaller size as compared to orotracheal intubation is commonly used for nasotracheal intubation. While tracheal tubes with 8.0-mm and 7.5-mm IDs are generally used for orotracheal intubation in adult males and females, respectively [9], NTTs of <7.0-mm ID are often used for nasotracheal intubation [10–13]. Yet, the effects of size, shape, and partial compression of the NTT in the nasal cavity on airway resistance have not been explored.

We hypothesized that use of a small NTT for nasotracheal intubation and/or partial compression of the NTT in the nasal passage may lead to a considerable increase in airway resistance. The aim of this study was to clarify the effects of size, shape, and partial compression of the NTT on airway resistance using physical and fluid dynamics simulation models. For this, the minor axis of the NTT in the nasal cavity of anesthetized patients was first measured to predict the compression pressure exerted by the nasal septum and nasal concha on the NTT; next, values of airway resistance with partial compression of tubes of various sizes were measured on the physical simulation model and calculated using established fluid dynamic equations.

## Methods

This study was carried out in accordance with the Declaration of Helsinki. It was conducted with the approval of the Medical Ethics Committee of Kindai University Faculty of Medicine (Ref: 26-230) and registered with the University Hospital Medical Information Network (UMIN UMIN000016942). The experiments for this study were carried out at the Product Development Research Center of Daiken Medical Co. Ltd., Osaka, Japan.

### Measurement of deformation of nasotracheal tubes in the nasal cavity

In the subsequent physical and fluid dynamics models, partial compression of the NTTs was simulated from the in vivo data. To extrapolate intranasal compression pressure from deformation of the NTT in the nasal cavity, the minor axis of the compressed NTT in the nasal cavity was measured in vivo, and the length of the nasal concha was measured on the axial CT image.

After written, informed consent was obtained from the patients, measurements of the minor axis of the NTT in the nasal cavities of the patients were performed at Kindai University Hospital. Between April and July 2015, 24 ASA physical status I or II patients, older than 18 years, who underwent elective oral and maxillofacial surgery in which nasotracheal intubation was required for surgical access were recruited. Exclusion criteria were severe obesity, anticipated difficult intubation, medication with anticoagulant drugs, frequent nasal obstruction, bleeding diathesis, and evident deviated nasal septum or deformity of the nasal concha on computed tomography (CT) images.

An experienced board-certified dental anesthesiologist performed all intubations. PVC nasal preformed tubes (Mallinckrodt™ Nasal RAE Endotracheal Tube with TaperGuard™ Cuff, Covidien Japan, Tokyo, Japan), sized 7.5-mm ID for men and 7.0-mm ID for women, were used for nasotracheal intubation. The thermo-softened NTT [12, 14, 15] was deliberately advanced through the right nostril into the trachea. If any resistance was felt, the procedure was re-attempted using the other nostril. Anesthesia was maintained with 1.5–2% sevoflurane in 50% oxygen balanced with nitrogen and a continuous intravenous infusion of remifentanil (0.25 μg/kg/min). After completion of surgery, the tube was cut at 3 cm above the nostril, and patients were ventilated with 100% oxygen through the reconnected slip joint and were continued to administer intravenous remifentanil; the minor axis of the tube in the nasal cavity was measured.

**Fig. 1:**
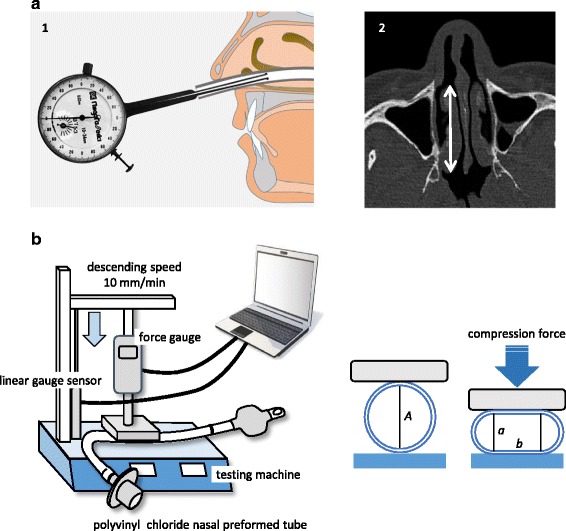
**a**-1 Schematic drawing of the clinical measurement of the internal minor axis of the nasotracheal tube. **a**-2 The length of the nasal concha (arrow) on the axial CT images. **b** Apparatus for measurement of pressure loss caused by deformation of the tubes. *A*: internal diameter of the uncompressed tube, *b*: length of the flat part of the compressed tube

An inside dial caliper gauge (BI-0, Niigata Seiki, Niigata, Japan), whose legs were processed to perform measurements over a range of 3–10 mm, was used for measurement of the minor axis of the NTT. The minor axis of the tube in the nasal cavity was measured by inserting the legs of the caliper gauge into the tube to a depth of 5 cm; thus, the minor axes of the tubes were measured at a depth of 2 cm inside the nostril. (Fig. 1a-1) After the measurement, anesthetic administration was discontinued, and the patient was extubated.

**Fig. 2:**
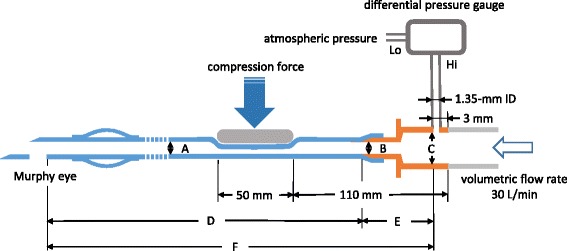
Schematic drawing of the measurement of pressure loss caused by deformation of the tubes. A: internal diameter of the endotracheal tube. B: internal diameter of the outlet of the slip joint, C: internal diameter of the inlet of the slip joint, D: length of the tubular part, E: length of the slip joint (25.5 mm), F: measured length of the endotracheal tube (D + E)

On the axial CT images of the patients taken for preoperative examination, the length of the nasal concha at 5–10 mm above the nasal base plane was obtained. (Fig. 1a-2).

### Prediction of compression force on the nasotracheal tubes in the nasal cavity

Based on the in vivo measures described above, the relationship between compression force and deformation of 6.0-, 6.5-, 7.0-, 7.5-, and 8.0-mm ID PVC NTTs was examined on in vitro benchtop tests. For this, a testing machine (LSC-1/300–2, Tokyo Koki Testing Machine Co. Ltd., Tokyo, Japan) was placed in the environmental test chamber (PR-3KPH, ESPEC Corp., Osaka, Japan) set at a temperature of 37 °C and relative humidity of 20%. A digital force gauge (DS2-200 N, Imada Co. Ltd., Aichi, Japan) and a linear gauge sensor (LGF-150 L, Mitutoyo Corp., Kanagawa, Japan) were attached to the testing machine. The tubes, which were laid on the table of the testing machine, were compressed by an acrylic plate 50 mm long and 30 mm wide, attached to the lower face of the cross head of the digital force gauge, with a 10 mm/min descending speed, until the tubes were compressed to a minor axis of 3 mm (Fig. 1b).

The values of the compression forces applied and the resultant minor axes of the NTTs were continuously recorded at intervals of 0.2 s during the experiments. The average values of compression force and minor axis obtained from five trials for each NTT size were calculated, and the relationship between the minor axis and the compression force was estimated. Based on the relationship between compression force and NTT deformation, the intranasal compression force was estimated.

### Pressure loss due to partial compression of nasotracheal tubes in the physical simulation model

Pressure losses caused by partial compression of the NTTs to a minor axis of 3 mm were measured in benchtop tests. To measure pressure loss through the NTT, measurement of the difference in pressure between the proximal end and the tip of the NTT was applied [2, 16–18]. Pressure losses through the NTTs were measured as described in our previous report [2].

The specifications of the nasal preformed PVC NTTs used in this study are shown in Table 1. Figure 2 shows a schematic drawing of the simulation model. Air flow into the tube or slip joint through the needle valve (Model 2412D, Kojima Instruments Inc., Kyoto, Japan) was regulated by the air regulator (RS-4-1, Fujikura Rubber Ltd., Tokyo, Japan), and the flow rate was monitored with a separate amplifier type air flow sensor (FD-V40A, KEYENCE Corp., Osaka, Japan). All measurements were performed under conditions of steady-state flow. Pressure loss through the partially compressed tube was measured using a differential pressure sensor (JP208-DFC, Yokogawa Electric Corp., Tokyo, Japan). In the in vivo study mentioned above, a section of the NTT approximately 11 to 16 cm from the proximal end was positioned within the nasal cavity. Hence, in this experiment, the portion of the tube between 11 and 16 cm from the proximal end of the tube was compressed by an acrylic plate 50 mm long and 30 mm wide, which corresponded to the length of the nasal concha. Since the volumetric flow rate of inspiratory airflow was estimated at 0.0005 m^3^/s (30 L/min) when a tidal volume of 500 mL, respiratory rate of 20 breaths/min, and inspiratory-to-expiratory time ratio of 1:2 were postulated, air flow was regulated at a rate of 30 L/min. The serial pressure loss through the NTT with every 0.5 mm of compression, until a minor axis of 3 mm was achieved by compression, was measured five times for each tube size.

**Table 1:** Specifications of the nasal preformed PVC tracheal tubes

	Length	Length of tubular part	Internal diameter	Wall thickness
6.0-mm ID	360.0 mm	332.8 mm	6.0 mm	1.1 mm
6.5-mm ID	370.0	342.2	6.5	1.1
7.0-mm ID	380.0	354.5	7.0	1.25
7.5-mm ID	390.0	359.8	7.5	1.3
8.0-mm ID	400.0	364.5	8.0	1.4

PVC: polyvinyl chloride, Length of tubular part: the length between the distal end of the slip joint and Murphy eye at the distal end of the tube. The length and internal diameter of inlet of the slip joints for all sized NTTs were 35.5 and 11.6 mm, respectively

The dimensions of the slip joints of the NTTs used in this study were the same as those of the standard tubes in our previous study. According to our previous report [2], pressure losses of 220.3, 131.1, 86.8, 57.1, and 36.1 Pa were used for the mean pressure losses through the slip joint for 6.0-, 6.5-, 7.0-, 7.5-, and 8.0-mm ID tubes, respectively. Hence, the difference between the total pressure loss (F in Fig. 2) and the pressure loss through the slip joint (E) was assumed to be the pressure loss of the tubular part (D) in this study.

### Predicted pressure loss due to partial compression of nasotracheal tubes in the fluid dynamics simulation model

The pressure losses through the tubular part of the NTT and those caused by compression of the variously sized tubes to a minor axis of 3 mm were calculated using the Darcy–Weisbach equation [19], and those through the slip joint, the bend in the NTT and the compressed part were estimated by measurement or calculation.

The lengths of the tubular parts of NTTs are shown in Table 1. In this fluid dynamics simulation, it was postulated that the tracheal tube was a straight and uniform tube, and that the portion of the tube between 11 and 16 cm from the proximal end of the tube was compressed in the nose.

The Reynolds Number (*Re*), which depends on the pipe diameter or equivalent diameter which is the diameter of a circular tube that gives the same pressure loss in non-circular pipe, was used to determine whether a flow would be laminar or turbulent: *Re* < 2000 indicated laminar flow, 2000 ≤ *Re* < 4000 indicated transient flow, and *Re* ≥ 4000 denoted turbulent flow [20]. The pressure loss through the compressed NTT depends on several factors: the uncompressed and partially compressed parts of the tubular part, the slip joint, and the curvature of the tube. Pressure losses through the tubular parts were predicted as the sum of the pressure losses in the uncompressed and compressed parts, calculated by the formulae described in the Appendix. The pressure losses through the slip joints reported in our previous study [2] were used for calculations in this study. The pressure loss through the curvature of the NTT, which was unchanged by partial compression, was assumed to be the difference between the measured and predicted pressure losses through the tubular parts of uncompressed NTTs.

The friction loss due to production of eddies in the airflow around the partial compression part was estimated as the discrepancy between the measured and predicted pressure losses through the tubular parts of the NTTs. Thus, the change in pressure loss through the NTT due to partial compression was the sum of the pressure loss through the partial compression part and the friction-related loss in pressure.

### Statistical analysis

Data are presented as means ± standard deviation for parametric data and medians with range for non-parametric data. Pressure loss is presented in Pa. Comparisons between groups were analyzed by the Mann-Whitney test. Data were analyzed using Prism software 5.0 (Graphpad, La Jolla, CA, USA). A value of *p* < 0.05 was considered significant.

## Results

### Measurement of deformation of nasotracheal tubes in the nasal cavity

Three patients were excluded from this study since they had deviated nasal septa. Hence, a total of 21 patients (11 men and 10 women) were assessed (Table 2). All tubes were successfully passed through the first nostril attempted. The median length of the nasal concha on CT images was 45.0 (31.8–54.5) mm at 5–10 mm above the nasal base plane.

**Table 2:** Subjects’ demographic data

	All subjects (*N* = 21)	Male (*N* = 11, 7.5-mm ID)	Female (*N* = 10, 7.0-mm ID)	*p*-value
Age (yr)	60.5 (18–82)	62.5 (38–82)	59.5 (18–70)	0.4955
Height (cm)	161.0 (141.7–177.9)	168.2 (155.0–177.9)	150.0 (141.7–166.5)	0.0007
Weight (kg)	56.4(43.9–80.4)	65.7 (43.9–80.4)	52.2 (47.8–60.4)	0.0036
BMI	23.5 (18.3–26.0)	23.1 (18.3–25.4)	23.7 (19.3–26.0)	0.5787
Length of nasal concha (mm)	45.0 (31.8–54.5)	45.9 (31.8–54.5)	44.7 (37.0–54.2)	0.9682

Median (range), *p*-value: 7.5-mm ID vs 7.0-mm ID tubes (Mann Whitney test), BMI: Body Mass Index

**Fig. 3:**
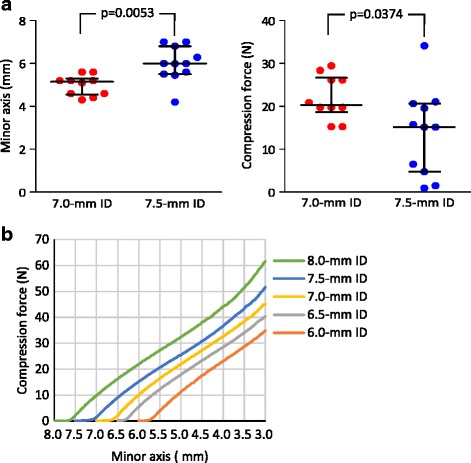
**a** Clinically measured minor axis (left) and predicted compression force (right) of 7.0- and 7.5-mm ID tubes in the nasal cavity by physical simulation. **b** Relationship between the minor axis of the tracheal tubes and external compression force. The lines indicate medians with interquartile range

The median minor axis of the tube at approximately 20 mm into the nasal cavity was 5.2 (4.3–5.6) mm in female patients using a 7.0-mm ID tube and 6.0 (4.2–7.0) mm in male patients using a 7.5-mm ID tube (Fig. 3aleft panel).

### Prediction of compression force on the nasotracheal tubes in the nasal cavity

In tubes of all sizes, the minor axis decreased by 0.3–0.5 mm with compression of less than 1 N, and then decreased linearly to 3 mm with further compression (Fig. 3b). This relationship between the minor axis and compression force indicated that predicted intranasal compression forces at the median minor axis of 5.2 (4.3–5.6) mm for 7.0-mm ID and 6.0 (4.2–7.0) mm for 7.5-mm ID tubes were 20.3 (15.3–29.4) N and 15.2 (0.9–34.1) N, respectively (Fig. 3a right panel). A maximum compression force of 34.1 N was predicted as being required to reduce the minor axis of 6.0-, 6.5-, 7.0-, 7.5-, and 8.0-mm ID tubes to 3.0, 3.5, 3.9, 4.2, and 4.8 mm, respectively.

### Pressure loss due to partial compression of nasotracheal tubes in the physical simulation model

**Fig. 4:**
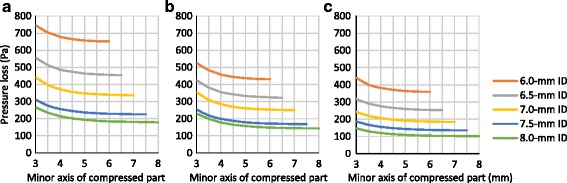
Total pressure losses and pressure losses through tubular parts of the nasotracheal tubes resulting from compression. **a** Total measured pressure losses through the nasotracheal tubes including the slip joint by physical simulation. **b** Predicted pressure losses through tubular parts, which is the total measured pressure loss minus the slip joint pressure loss, by physical simulation. **c** Calculated pressure losses through the tubular parts by fluid dynamics simulation

With a volumetric air flow rate of 30 L/min, total pressure losses through 6.0-, 6.5-, 7.0-, 7.5-, and 8.0-mm ID uncompressed NTTs were 651.6 ± 5.7, 453.4 ± 3.9, 336.5 ± 2.2, 225.2 ± 0.2, and 179.0 ± 1.1 Pa, respectively; pressure losses through the tubular parts, which were the total measured pressure losses minus the previously reported slip joint pressure loss, of each uncompressed NTT were 431.3 (66.2% of total pressure loss), 322.2 (71.1%), 249.7 (74.2%), 168.0 (74.6%), and 143.0 Pa (79.9%), respectively. Pressure loss through the NTTs increased non-linear polynomially with reduction in the minor axis, roughly by less than 10 Pa at a minor axis of more than 5 mm, by more than 30 Pa at a minor axis of 4 mm, and by 100 Pa at a minor axis of 3 mm, compared to the pressure losses in the uncompressed NTTs of each size. (Fig. 4a, b) With a compression force of 34.1 N, total pressure losses through 6.0-, 6.5-, 7.0-, 7.5-, and 8.0-mm ID compressed NTTs at the minor axis of 3.0, 3.5, 3.9, 4.2, and 4.8 mm of compression part, respectively, were calculated by interpolation between measured data points as 745.8 (114.5% of the uncompressed tube), 513.0 (113.2%), 372.2 (110.6%), 251.6 (111.7%), and 196.7 Pa (109.9%), respectively.

### Predicted pressure loss due to partial compression of nasotracheal tubes in the fluid dynamics simulation model

The equivalent diameter, which was calculated by the formulae described in the Appendix, ranged from 4.50 mm (minor axis of 3 mm in a compressed 6.0-mm ID tube) to 8.0 mm (an uncompressed 8.0-mm ID tube). Since *Re* was calculated as being >4000 (range 5305–9431), airflow through the tubes was expected to be turbulent rather than laminar [20].

Calculated pressure losses through non-curved 6.0-, 6.5-, 7.0-, 7.5-, and 8.0-mm ID uncompressed tubular parts were 359.6, 253.3, 184.9, 135.6, and 101.3 Pa, respectively. The pressure losses through the partially compressed parts of each tube were predicted to increase by approximately 10 Pa at a minor axis of more than 5 mm and approximately 20 Pa at a minor axis of 4 mm; the predicted pressure losses through NTTs of 6.0-, 6.5-, 7.0-, 7.5-, and 8.0-mm ID compressed to a minor axis of 3 mm were 82.0, 64.6, 57.5, 51.3, and 45.8 Pa larger than those in the corresponding uncompressed NTTs of each size, respectively. Pressure losses through compressed 6.0-, 6.5-, 7.0-, 7.5-, and 8.0-mm ID NTTs with minor axes of 3.0, 3.5, 3.9, 4.2, and 4.8 mm, respectively, resulting from application of the maximum intranasal compression force of 34.1 N, were calculated as 82.0, 38.0, 23.5, 16.6, and 9.3 Pa, respectively (Fig. 4c).

**Fig. 5:**
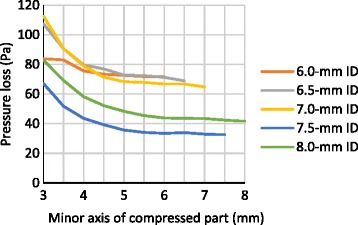
Calculated pressure losses due to the curvature of the nasotracheal tubes resulting from compression

The pressure loss due to the curvature of the NTTs in 6.0-, 6.5-, and 7.0-mm ID tubes, which was the difference between measured and calculated pressure loss in a straight tube, was approximately 70 Pa, and those in NTTs of 7.5- and 8.0-mm IDs were 30 and 40 Pa, respectively. Friction loss around the partial compression part increased non-linear polynomially with reduction in the minor axis (Fig. 5).

## Discussion

In this study we demonstrated that pressure loss through NTT was greater than through a standard oral endotracheal tube at sizes of 6.0, 6.5, 7.0, 7.5 and 8.0-mm ID. We found that diameter was the largest factor in NTT pressure loss, with a 6.0 mm NTT having more than 3 times the pressure loss of an 8.0 mm NTT at air flow rates of 30 L/min. The slip joints and the acute curvature of NTT contributed to approximately 20–35% and 10–15% of this pressure loss, respectively, and were worse in smaller tube diameters. External compression of the NTT similar to in the nasal cavity further increased the NTT pressure loss by approximately 5–10%, with a larger absolute increase for smaller NTT.

### Methodology for measurement and estimation of pressure loss through the tracheal tube

To measure pressure loss through the tracheal tube, two kinds of measurements have been used in previous reports: measurement of the difference in pressure between the proximal end of the tracheal tube and the tip of the tube in the trachea [21–24], and measurement of the difference in pressure between the proximal end of the tracheal tube and the tip of the tracheal tube open to the atmosphere [2, 16–18]. Although the former measurement has been applied in tracheal models and clinical measurements, it is influenced by pressure changes due to a sudden increase in fluid flow at the tip of the tracheal tube in the trachea [21, 25]. In this study, the latter measurement method was applied to measure pressure loss through the NTT to eliminate the influence of a sudden increase in fluid flow at the tip of the tracheal tube in the trachea.

Although Bernoulli’s equation states that the sum of the kinetic, potential, and flow energies of a fluid particle is constant when compressibility and viscosity are negligible and flow is steady or laminar, there is still energy loss due to the friction in any pipe system. In a long straight section of a round pipe, the Darcy–Weisbach equation [19] is able to calculate the energy loss due to friction (major loss) for both laminar and turbulent flows. The pressure loss from flow separation and mixing due to a bend in the pipe, sudden expansion or contraction of the pipe, or a different pipe fitting is considered a minor loss [25, 26]. The total pressure loss through the pipe consists of the sum of major and minor losses. In this study, the major losses in the tubular part of the NTT were calculated using the Darcy–Weisbach equation [19], and minor losses in the components, namely, the slip joint, the bend in the NTT, and the compressed part, were estimated by measurement or calculation.

### Contribution of components of the nasotracheal tube to respiratory pressure loss

It has been recognized that the size of the endotracheal tube is the major factor governing resistance in anesthesia breathing systems [1]. In standard endotracheal tubes, the pressure loss through the tubular part depends on the internal diameter, length, and volumetric air flow rate, while the effect of preformed curvatures on airway resistance is minimal [2, 27, 28]. Furthermore, the pressure loss through the slip joint, which is considered a minor loss, accounts for approximately 25–40% of the total pressure loss, and the ratio of pressure loss through the slip joint to the total pressure loss is markedly larger in small than in large ID tubes [2].

Preformed NTTs also have other properties that contribute to increasing pressure loss as compared with standard endotracheal tubes. They are longer than standard tracheal tubes, have a sharp bend along the bridge of the nose, and get compressed in the nasal cavity. The overall pressure loss through the partially compressed preformed NTT in the nasal cavity, therefore, consists of pressure losses through the uncompressed tubular part, the slip joint, the sharp bend of the NTT, and the partly compressed tubular part.

**Fig. 6:**
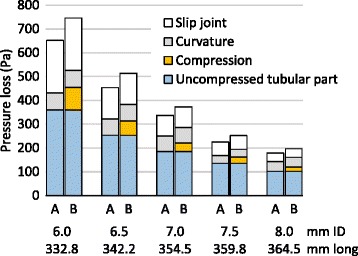
Factors involved in increasing the pressure losses through nasotracheal tubes with a maximum intranasal compression of 34.1 N at a flow rate of 30 L/min in the physical and the fluid dynamics simulations. A: uncompressed tubes, B: partially compressed tubes with maximum compression force

Although NTTs with smaller IDs are shorter than those with larger IDs, the measured pressure losses through tubes of small ID are much greater than those through tubes of large ID. In standard endotracheal tubes with a slight curvature, the predicted pressure losses through tubular parts, as calculated using the equation for turbulence, are reported approximately equal to the measured values [2, 18]. In this study using preformed NTTs, the measured pressure losses through the tubular part, especially in NTTs of small ID, were obviously larger than the predicted values (Fig. 6). The pressure loss due to a bend is dependent on the ratio of the radius of curvature of the bend to the diameter of the pipe [29]. We surmised that the discrepancies in pressure loss through the tubular part of NTTs between measured and predicted values are mainly due to the sharp bend of the NTTs, and that the pressure losses due to the bend increase in the preformed NTTs with smaller IDs. In this study, the pressure losses through the slip joints constituted 20–35% of the total pressure losses through uncompressed preformed NTTs. These additional features in NTTs, such as sharp bends and slip joints, cause obvious increases in the pressure loss, which are unaffected by partial compression of the NTT.

In the nasal cavity, NTTs of 7.0- and 7.5-mm ID were compressed by median compression forces of 20.3 and 15.2 N, respectively. In other words, intranasal pressure in women, who were significantly shorter and lighter than men, seemed to be higher than in men. Increasing intranasal compression forces could reduce the minor axis and increase pressure loss. In this study, the maximum intranasal compression force of 34.1 N during nasotracheal intubation increased pressure losses by 82.0 Pa (0.8 cmH_2_O) in the 6.0-mm ID NTT, but 9.3 Pa (0.1 cmH_2_O) in the 8.0-mm ID NTT. Thus, increasing pressure loss due to partial compression of the NTT by intranasal compression cannot be ignored, especially in smaller ID NTTs.

The present study has certain limitations. First, the actual compression force could be different from our estimates, since the NTT might be compressed unequally by mucosa lining the nasal concha and nasal septum in the nasal cavity. Furthermore, the NTT, especially in a large sized NTT, could be pressured by large forces from a bony ridge in the nasal cavity. Although we measured pressure loss due to partial compression of the NTT using the simulation model with equal compression forces obtained with a plastic plate, more accurate pressure loss would be obtained by a simulation model with compression by an anatomically shaped object made of an elastic material. Second, pressure losses were evaluated at a volumetric flow rate of 30 L/min. The flow rate can increase up to 60–100 L/min in patients with high inspiratory flow demands [30]. A high flow rate generates eddies in the flow and further increases the Reynolds number and pressure loss through the tube.

## Conclusions

In conclusion, the results of the present study indicate that pressure losses through smaller-sized NTTs are greater than those through larger-sized NTTs. Although larger-sized NTTs may increase the risk of epistaxis, from the perspective of the work of breathing, the use of larger-sized NTTs should be considered in patients that require nasotracheal intubation, especially among patients with abnormal respiratory mechanics that may require larger mechanical support during or weaning from mechanical ventilation.

## Appendix

### Calculation of the pressure losses through a non-curved tubular parts with partial compression

With external compression of the tubes, the cross-sections of the tubes were assumed to approximate a flat oval shape, equal to a rectangle with semicircles at both ends. Equivalent diameter (*De*), which is the diameter of a circular tube that gives the same pressure loss as a corresponding flat ovalshaped tube, was calculated as follows [31]:

1 $$ \mathrm{L}=\uppi \mathrm{D}=\uppi \mathrm{a}+2\mathrm{b}\kern0.75em $$

2 $$ \mathrm{b}=\frac{\pi \left(D-a\right)}{2}\kern0.75em $$

3 $$ De=1.55\times \frac{{\left\{\pi {a}^2/4+a\left(a+b\right)-{a}^2\right\}}^{0.625}}{{\left\{\pi a+2\left(a+b\right)-2a\right\}}^{0.25}} $$

4 $$ De=1.55\times \frac{{\left[\pi {a}^2/4+a\left\{a+\pi \left(D-a\right)/2\right\}-{a}^2\right]}^{0.625}}{{\left[\pi a+2\left\{a+\pi \left(D-a\right)/2\right\}-2a\right]}^{0.25}} $$

where L is the circumference of the tube, D is the internal diameter of the uncompressed tube, a is the minor axis of the compressed tube, and b is the length of the flat part of the compressed tube.

The pressure losses in the tubular parts were estimated as follows. The values for density of air (*ρ*), coefficient of viscosity of air (μ), and absolute surface roughness height of the tube (ε) were 1.2 kg/m³ (20°C), 0.000018 Pa⋅s (20°C), and 0.0015 mm, respectively [19]. The volumetric flow was set at 30 L/min. the friction factor (f) was calculated according to the Swamee-Jain equation approximated to the Colebrook equation [32], and pressure loss through the tubes (*∆P*) was estimated by the Darcy-Weisbach equation as follows [19]:

5 $$ \mathrm{u}=\mathrm{V}/\left(\uppi {De}^2/4\right) $$

6 $$ \mathit{\operatorname{Re}}=\uprho \mathrm{u} De/\upmu $$

7 $$ \mathrm{f}=\frac{0.25}{{\left[\log \kern0.5em \left(\upvarepsilon /3.7 De+5.74/{Re}^{0.9}\right)\right]}^2} $$

8 $$ \Delta  P=\mathrm{f}\left(\frac{{\rho u}^2}{2}\right)\left(\frac{L}{De}\right) $$

where u is the mean velocity of the flow (m/s), V is the volumetric flow rate (m^3^/s), *De* is the equivalent diameter (m), *Re* is the Reynolds number, ρ is the density of the fluid (kg/m³), μ is the coefficient of viscosity (Pa⋅s), f is the friction factor, ε is the absolute surface roughness height (m), L is the pipe length (m), and *∆P* is the pressure loss (Pa).

## Abbreviations

*D*e: Equivalent diameter
ID: Internal diameter
NTT: Nasotracheal tube
PVC: Polyvinyl chloride
*Re*: Reynolds Number
